# Mining local and global spatiotemporal features for tactile object recognition

**DOI:** 10.3389/fnbot.2024.1387428

**Published:** 2024-05-03

**Authors:** Xiaoliang Qian, Wei Deng, Wei Wang, Yucui Liu, Liying Jiang

**Affiliations:** College of Electrical and Information Engineering, Zhengzhou University of Light Industry, Zhengzhou, China

**Keywords:** tactile object recognition, LGR-18 network, local convolution module, global convolution module, local and global spatiotemporal features

## Abstract

The tactile object recognition (TOR) is highly important for environmental perception of robots. The previous works usually utilize single scale convolution which cannot simultaneously extract local and global spatiotemporal features of tactile data, which leads to low accuracy in TOR task. To address above problem, this article proposes a local and global residual (LGR-18) network which is mainly consisted of multiple local and global convolution (LGC) blocks. An LGC block contains two pairs of local convolution (LC) and global convolution (GC) modules. The LC module mainly utilizes a temporal shift operation and a 2D convolution layer to extract local spatiotemporal features. The GC module extracts global spatiotemporal features by fusing multiple 1D and 2D convolutions which can expand the receptive field in temporal and spatial dimensions. Consequently, our LGR-18 network can extract local-global spatiotemporal features without using 3D convolutions which usually require a large number of parameters. The effectiveness of LC module, GC module and LGC block is verified by ablation studies. Quantitative comparisons with state-of-the-art methods reveal the excellent capability of our method.

## 1 Introduction

Robots perceive objects around them mainly through touch and vision. Although vision can intuitively capture the appearance of an object, it cannot capture the basic object properties, such as mass, hardness and texture. In addition, many limitations exist for vision perception (Lv et al., 2023a). Consequently, the tactile object recognition (TOR) task is proposed to predict the category of the object being grasped by robot, which can provide support for subsequent grasping operations without being constrained by the aforementioned conditions.

TOR has a broad range of applications in life, including descriptive analysis in the food industry (Philippe et al., 2004), electronic skin (Liu et al., 2020) and embedded prostheses in the biomedical field (Wu et al., 2018), and postdisaster rescue (Gao et al., 2021), etc. The rapid development of deep learning (Lv et al., 2023c) has led to tremendous progress in various fields (Qian et al., 2020, 2023a,b,d,e,f; Huo et al., 2023; Li et al., 2023; Xie et al., 2024), and deep learning based TOR are the mainstream methods (Liu et al., 2016; Ibrahim et al., 2022; Yi et al., 2022). Currently, TORs using deep learning can be divided into two categories: one uses a 2D CNN to extract features from each frame of tactile data and then fuses the features of each frame to recognize the grasped object based on the fused features and the other uses a 3D CNN to extract spatial and temporal features from tactile frames for recognition.

Traditional TOR methods mostly adopt the methods in the first category, which use sensors (primarily pressure sensor arrays) to acquire tactile information, then the tactile data are sent to a 2D CNN for feature extraction and category prediction. Gandarias et al. (2017) used a 2D CNN to extract high-resolution tactile features and trained a support vector machine (SVM) using these features. The trained SVM was used to predict the object category. Bottcher et al. (2021) collected tactile data via two different tactile sensors and subsequently input the data into a 2D CNN to extract features and infer results. Other related works include Sundaram et al. (2019), Chung et al. (2020), and Carvalho et al. (2022), etc.

Recently, the another category of methods has achieved remarkable results and has become mainstream. Qian et al. (2023c). used a gradient adaptive sampling (GAS) strategy to process the acquired tactile data and subsequently fed the data into a 3D CNN network to extract multiple scale temporal features. The features were fused at the fully connected and outputted prediction category. Inspired by the optical flow method (Cao et al., 2018) used not only original tactile data but also tactile flow and intensity differences as input data. These data underwent convolution, weighting, and other operations on different branches and were ultimately fused at the fully connected layer to infer the results. Other related works include Kirby et al. (2022) and Lu et al. (2023), etc.

The first category of methods extracts only the features of each frame and does not use temporal information between frames, therefore, their overall performance is limited. In the second category, spatiotemporal features are extracted via 3D CNNs, and the overall performance is better than that of the first category of methods. However, existing methods utilize single scale convolution operations to extract features in spatial and temporal dimensions, and they cannot simultaneously extract local and global features very well.

This paper proposes a local-global spatiotemporal feature extraction scheme to solve the above problems. First, a local convolution (LC) module is proposed, which utilizes the interaction of adjacent temporal information and 2D convolution to extract local spatiotemporal features. Next, the global convolution (GC) module is proposed to extract global spatiotemporal features and the module combines multiple 1D and 2D [(1+2)D] convolutions which can extend the receptive field (Lv et al., 2023b) in spatial and temporal dimensions. Finally, this article achieves accurate TOR by comprehensively utilizing local and global spatiotemporal features.

The main contributions are as follows:

1. This paper proposes a local convolution (LC) module which extracts spatiotemporal features by using the interaction of adjacent temporal information and 2D convolution operation.

2. This paper proposes a global convolution (GC) module which extracts global spatiotemporal features by fusing multiple 1D and 2D convolutions which can expand the receptive field in temporal and spatial dimensions.

3. Our method achieves the highest object recognition accuracy on two public datasets by comprehensively using local-global spatiotemporal features.

## 2 Related works

Gradient adaptive sampling (GAS) (Qian et al., 2023c) and MR3D-18 network (Qian et al., 2023c) have strong relevance to this paper, therefore, they are introduced here.

### 2.1 Gradient adaptive sampling

Unlike uniform sampling and sparse sampling, GAS uses the pressure gradient to guide the adaptive sampling. The specific approach is to normalize the accumulated gradient over the *T* period and then divide it into multiple intervals, randomly selecting one point from each interval. This process obtains multiple data frames, which are subsequently fed into the network.

### 2.2 MR3D-18 network

The MR3D-18 network is proposed to address the problems that the size of tactile frames is small and overfitting is easily occur, which removes a pooling operation and adds a dropout layer to the ResNet3D-18 (Hara et al., 2018) network for handling above problems.

## 3 Proposed method

### 3.1 Overview

**Figure 1:**
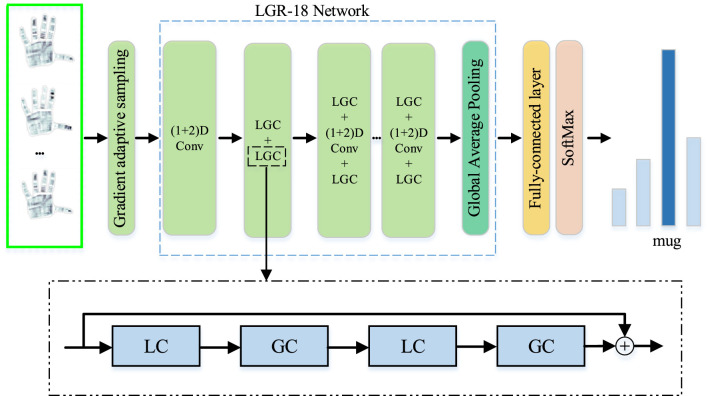
Framework of our method.

As shown in Figure 1, the P frames of tactile data are adaptively selected from all frames by using GAS. Then, they are fed into local and global residual (LGR-18) network to extract local and global spatiotemporal features. Finally, the features are imported into a fully connected layer and a softmax classifier to predict categories. Our LGR-18 network is primarily composed of multiple local and global convolution (LGC) blocks, which is proposed in this paper, and the LGC block consists of two pairs of LC and GC modules. The LC and GC modules, LGC block and LGR-18 network will be precisely explained in the following sections.

### 3.2 Local convolution module

**Figure 2:**
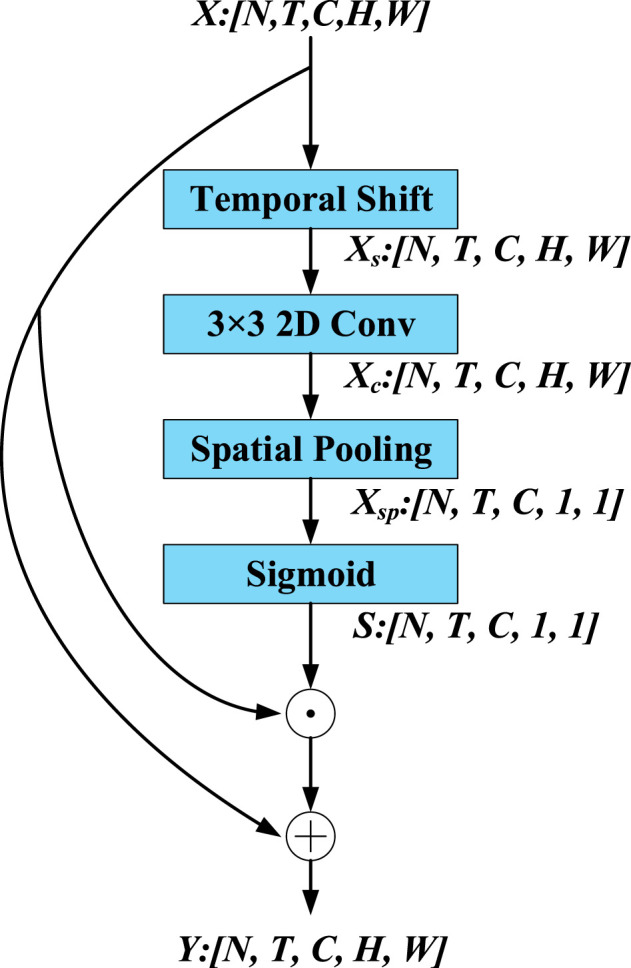
Illustration of LC module.

The LC module focuses on extracting the local spatiotemporal features of tactile data. As shown in Figure 2, the size of the input features *X* is [*N*, *T*, *C*, *H*, *W*], where *N* denotes the batch size, *T* and *C* separately denote the quantity of input frames and feature channels, *H* and *W* denote the height and width of the features respectively. First, the LC module utilizes the temporal shift (TS) operation to extract local temporal features.

**Figure 3:**
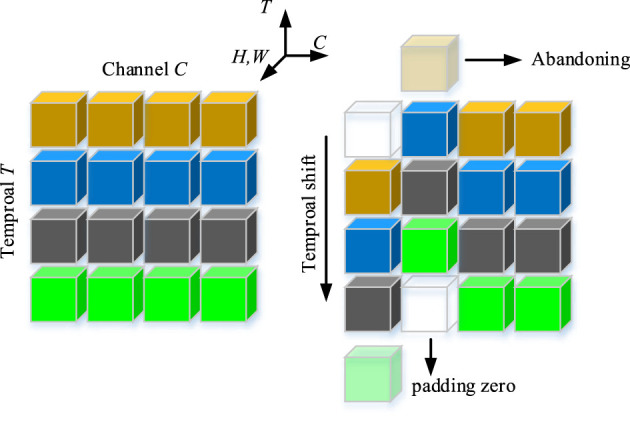
Illustration of temporal shift operation.

The TS operation is shown in Figure 3. A tensor with *C* channels and *T* frames is also shown in Figure 3. The features of the different time stamps are shown in different colors in each row. Along the temporal dimension, the TS operation shifts one channel in forward direction and one channel in backward direction. We utilize the abandoning and zero-padding operation to address the problems of excessive and missing features. It is worth noting that replacing the zero padding features by the abandoning features is infeasible because it will destroy the temporal sequence. After the TS operation, a 2D convolution layer is utilized to extract the local spatial features. Next, we utilize a global average pooling (GAP) and a sigmoid function to extract the local spatiotemporal weights of each channel. Finally, we utilize a simple method to extract local spatiotemporal features by performing channel-wise product between the input feature *X* and the local spatiotemporal weight *S*. A residual connection is employed to prevent the loss of crucial information in the original features. Finally, the LC module, which relies on TS operations and traditional 2D convolutions as its core, extracts local features.

### 3.3 Global convolution module

**Figure 4:**
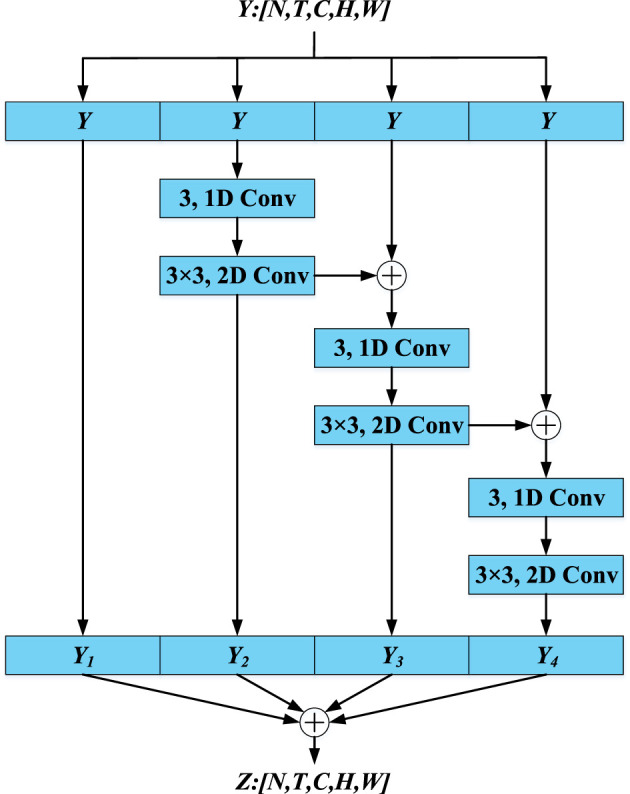
Illustration of GC module.

Inspired by the conventional depthwise separable convolution, multiple (1+2)D convolutions are utilized to extract global spatiotemporal features from tactile data, where the 1D and 2D convolutions are used to extract temporal and spatial features, respectively. However, the innovation of GC module does not lie in the depthwise separable convolution. As shown in Figure 4, the core idea of GC module is that the receptive field of convolution kernels is continuously enlarged to extract global spatiotemporal features via iterative residual connections and convolutions. The detailed procedure can be seen in Equations 1, 2 and Figure 4.


(1) $$\{\begin{array}{l} Y_{1}=Y\\ Y_{2}=conv_{(1+2)D}(Y)\\ Y_{3}=conv_{(1+2)D}(Y+Y_{2})\\ Y_{4}=conv_{(1+2)D}(Y+Y_{3}) \end{array}Y_{1},Y_{2},Y_{3},Y_{4}\in R^{N,T,C,H,W}$$

In Equation 1, *Y*_1_ is the output of the first branch and is identical to the input of the first branch. *Y*_2_, *Y*_3_ and *Y*_4_ represent the outputs of the 2nd, 3rd and 4th branches, respectively, in the GC module. The *conv*_(1+2)*D*_ is the same as (1+2)D convolutions. The parameters for *conv*_(1+2)*D*_ are 3 and 3–3. As shown in Equation 2, the final output of GC module, denoted as Z, is obtained by fusing the outputs of four branches:


(2) $$\begin{array}{l} Z=\overset{4}{\underset{i=1}{\sum }}Y_{i}\;Z\in R^{N,\;T,\;C,\;H\;,W} \end{array}$$

### 3.4 Architecture of the LGR-18 network

As shown in Table 1, our LGR-18 network is modified from the MR3D-18 network. First, in the LGR-18 network, the (1+2)D convolutions replaced the 7 × 7 × 7 convolution layer in the MR3D-18 network. Second, in the LGR-18 network, multiple 3 × 3 × 3 3D convolution layers are replaced with LGC blocks, which is proposed in this paper, and two 3D convolution layers are approximately equivalent to an LGC block. Third, the LGR-18 network adds (1+2)D convolutions with size of 1 in the Res_3_, Res_4_, and Res_5_ layers to change the number of channels. Finally, the LGC block consists of two pairs of LC and GC modules connected by residual connections. Table 1 shows that the LGR-18 network has two advantages over the MR3D-18 network.

**Table 1:** Comparison between MR3D-18 and LGR-18 network, where the size of input tactile data is 32 × 32 × 32.

**Layers**	**MR3D-18**		**LGR-18**	
	**Filters**	**Output size**	**Filters**	**Output size**
Conv_1_	7 × 7 × 7, 64 stride 1, 2^2^	32 × 16^2^×64	7 7 × 7 stride 1, 2^2^	32 × 16^2^×64
Res_2_	$\left[\begin{array}{c} 3\times 3\times 3,64\\ 3\times 3\times 3,64 \end{array}\right]\times 2$ stride 1,1^2^	32 × 16^2^×64	LGC, 64 LGC, 64 stride 1,1^2^	32 × 16^2^×64
Dropout	0.3	32 × 16^2^×64	0.3	32 × 16^2^×64
Res_3_	$\left[\begin{array}{c} 3\times 3\times 3,128\\ 3\times 3\times 3,128 \end{array}\right]\times 2$stride 2,2^2^	16 × 8^2^×128	LGC, 64 1, 128 1 × 1, 128 LGC, 128 stride 2,2^2^	16 × 8^2^×128
Res_4_	$\left[\begin{array}{c} 3\times 3\times 3,256\\ 3\times 3\times 3,256 \end{array}\right]\times 2$stride 2,2^2^	8 × 4^2^×256	LGC, 128 1, 256 1 × 1, 256 LGC, 256 stride 2,2^2^	8 × 4^2^×256
Res_5_	$\left[\begin{array}{c} 3\times 3\times 3,512\\ 3\times 3\times 3,512 \end{array}\right]\times 2$stride 2,2^2^	4 × 2^2^×512	LGC, 256 1, 512 1 × 1, 512 LGC, 512 stride 2,2^2^	4 × 2^2^×512
Global average pooling				

1. The LGR-18 network abandons all 3D convolution layers, reducing computational complexity.

2. The LGR-18 network can extract local and global spatiotemporal features through the LGC blocks.

### 3.5 Training scheme

To enhance the performance of the LGR-18 network, we utilize the large-scale Kinetics-400 dataset (Carreira and Zisserman, 2017), which consists of 400 human action categories and at least 400 video clips in each category, to pre-train our method. Subsequently, we utilized the UCF101 (Soomro et al., 2012) and target datasets to pretrain the LGR-18 network.

To address the problem of large dataset sizes, the size of input data is adjusted to 32 × 32 when the LGR-18 network pre-trained on the Kinetics-400 and UCF101 datasets. The traditional cross-entropy loss, denoted as *L*, is employed to optimize the LGR-18 network, which is formulated Equation 3:


(3) $$\begin{array}{l} L=-\overset{K}{\underset{k=1}{\sum }}v_{k}\log v_{k}^{\prime }\; \end{array}$$

where *K* denotes the number of categories, $v_{k}^{\prime }$ denotes the prediction score of category *k*, and *v*_*k*_ denotes the label of category *k*.

## 4 Experiment

### 4.1 Experiment setup

#### 4.1.1 Datasets

The LGR-18 network is verified on the MIT-STAG (Sundaram et al., 2019) and iCub datasets (Soh et al., 2012). The MIT-STAG dataset includes 26 categories of common objects and empty hands, i.e., allen key set, ball, battery, board eraser, bracket, stress toy, cat, chain, clip, coin, gel, kiwano, lotion, mug, multimeter, pen, safety glasses, scissors, screwdriver, spoon, spray can, stapley, tape, tea box, full cola can, and empty cola can. A total of 88,269 valid frames are collected, each with a size of 32 × 32. To achieve accurate prediction results under fair conditions, 1,353 frames (totaling 36,531 frames) are selected from each category as the training set, and 597 frames (totaling 16,119 frames) are selected as the testing set. The MIT-STAG dataset includes too many categories with similar appearance characteristics, making accurate object recognition highly challenging on this dataset.

The iCub dataset is acquired by two anthropomorphic dexterous hands of the iCub humanoid robot platform. Each anthropomorphic hand is equipped with five fingers, each with 20 movable joints. Additionally, each finger is equipped with pressure sensors to acquire tactile data. The iCub dataset includes 2,200 frames with 10 categories, i.e., monkey toy, med vitamin water, med coke, lotion, vitamin water, full cola, empty vitamin water, empty coke, book and blue bear (toy), and the size of each frame is 5 × 12. For each category in the iCub dataset, 132 frames (totaling 1,320 frames) are selected as the training set, and 88 frames are selected (totaling 880 frames) as the testing set.

#### 4.1.2 Implementation details

This paper uses the top 1 score, kappa coefficient (KC) and confusion matrix for evaluation. The stochastic gradient descent is used to optimize our module. The momentum and decay rate are 0.9 and 0.0001, respectively. The initial learning rate is 0.002 and the quantity of epochs is 50. The learning rate decreases to 10% of the previous stage after every 10 epochs. The batch sizes are 32 and 8 for the MIT-STAG and iCub datasets, respectively.

The experiments were all performed on the PyTorch framework and run on a workstation with two NVIDIA GeForce RTX 2080 Ti (2 × 11 GB).

### 4.2 Ablation study

#### 4.2.1 Ablation study of LGC block

As Table 2 shows, the LGC block is compared with the other four convolution blocks to verify its effectiveness. Architecture A is used as a baseline and is composed of two 3D convolution layers connected by residual connection. Architecture B replaces one of the 3D convolutions layers in architecture A with an LC module, and architecture C uses the GC module to replace one of the 3D convolutions layers in architecture A. In architecture D, a pair of LC and GC modules are utilized to replace one of two 3D convolution layers in architecture A. Architecture E is our method, and it uses two pairs of LC and GC modules to replace all the 3D convolution layers in architecture A.

**Table 2:** Ablation study of LGC block on the MIT-STAG dataset.

**Architecture**	**Illustration of architecture**	**Top 1 score**
A(baseline)	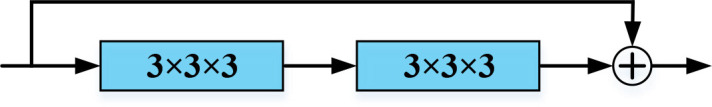	84.98
B	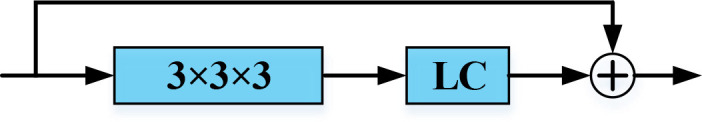	85.32
C	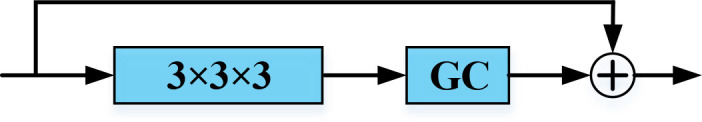	88.07
D	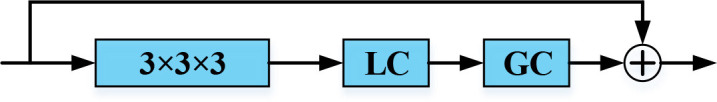	89.05
E	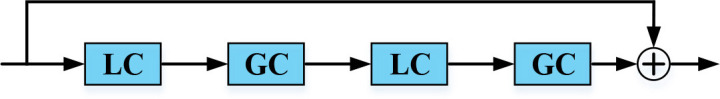	91.07

Ablation studies reveal the effectiveness of the LC module, GC module, and combination of the LC and GC modules to compare architectures B, C, and D with A. By comparing architecture E with A, ablation studies strongly demonstrate that the best performance can be achieved by using a combination of LC and GC modules.

#### 4.2.2 Ablation study of spatial pooling operation in LC module

As shown in Figure 2, the spatial pooling operation is involved in the LC module, therefore, the max pooling and GAP are compared with each other to determine who is more appropriate for LC module. As shown in Table 3, the top 1 score and KC of GAP are higher than the ones of max pooling, consequently, the GAP is adopted by LC module.

**Table 3:** Ablation study of LGC block on the MIT-STAG dataset.

**Spatial pooling operation**	**Top 1 score**	**KC**
Max pooling	90.8	89.7
Global average pooling	91.1	90.7

### 4.3 Parameter analysis

#### 4.3.1 Parameter analysis for the number of shifting channels

**Figure 5:**
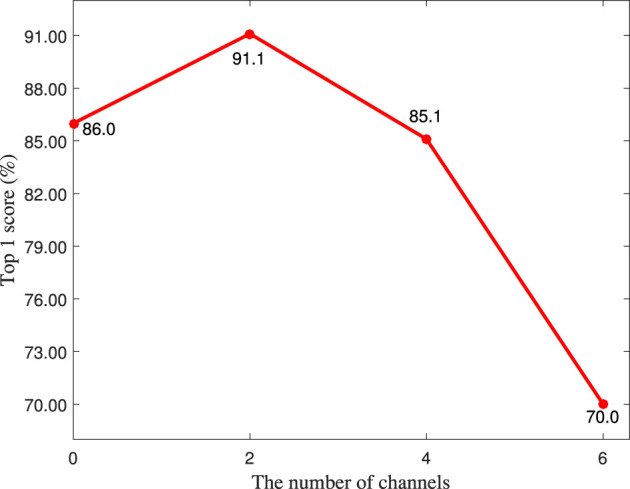
The top 1 score (%) of different number of shifting channels on the MIT-STAG dataset.

As shown in Figure 3, the number of shifting channels is an important hyperparameter for the LC module, therefore, it is quantitatively analyzed in this section. It is worth noting that the number of shifting channels must be even because the shifting operation is bidirectional. As shown in Figure 5, the top 1 score achieves the highest value when the number of shifting channels is set to 2, which means that shifting many channels is not suitable for the LC module because it can induce the information reduction.

#### 4.3.2 Parameter analysis for dropout rate

**Figure 6:**
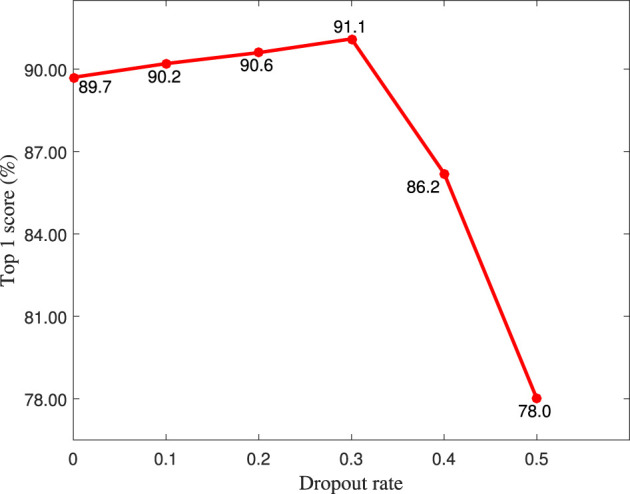
The top 1 score (%) of different dropout rate on the MIT-STAG dataset.

As shown in Table 1, the dropout layer is used to prevent the overfitting problem, therefore, the dropout rate is quantitatively analyzed in this section. As shown in Figure 6, the top 1 score achieves the highest value when the dropout rate is set to 0.3.

### 4.4 Comparisons with state-of-the-art methods

To demonstrate the overall effectiveness of our model, we conducted a comprehensive quantitative comparison with 5 methods on the MIT-STAG dataset (Sundaram et al., 2019), i.e., STAG (Sundaram et al., 2019), Smart-hand (Wang et al., 2021), ResNet10-v1 (Zhang et al., 2021), Tactile-ViewGCN (Sharma et al., 2022), and GAS-MR3D (Qian et al., 2023c), and 5 methods on the iCub dataset (Soh et al., 2012), i.e., DS (Soh and Demiris, 2014), GS (Soh and Demiris, 2014), STORK-GP (Soh et al., 2012), STAG (Sundaram et al., 2019), and GAS-MR3D (Qian et al., 2023c).

**Figure 7:**
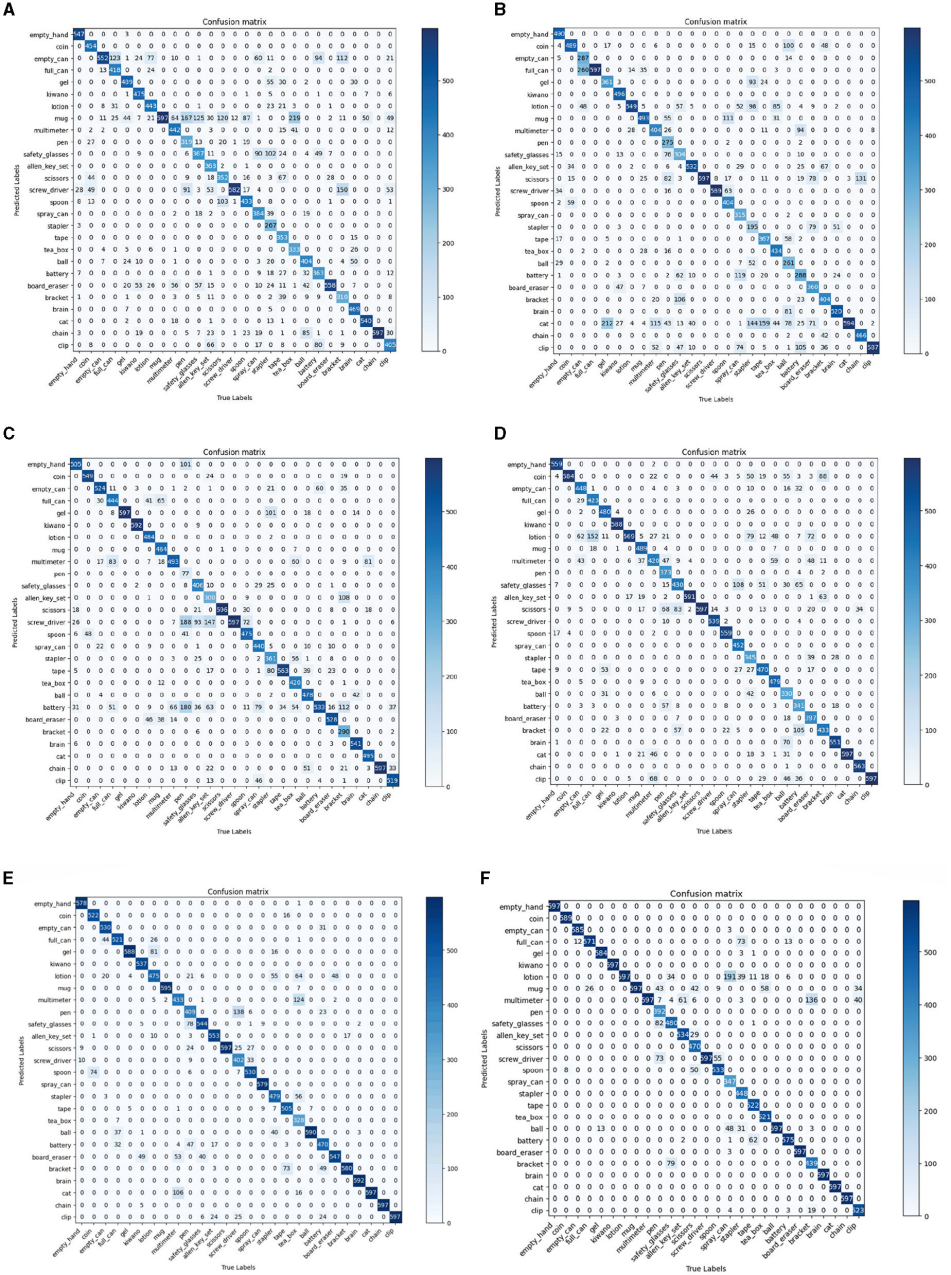
Comparisons with 5 methods in terms of confusion matrix on the MIT-STAG dataset. **(A)** STAG (Sundaram et al., 2019), **(B)** Smart-hand (Wang et al., 2021), **(C)** ResNet10-v1 (Zhang et al., 2021), **(D)** Tactile-ViewGCN (Sharma et al., 2022), **(E)** GAS-MR3D (Qian et al., 2023c), **(F)** Ours.

**Figure 8:**
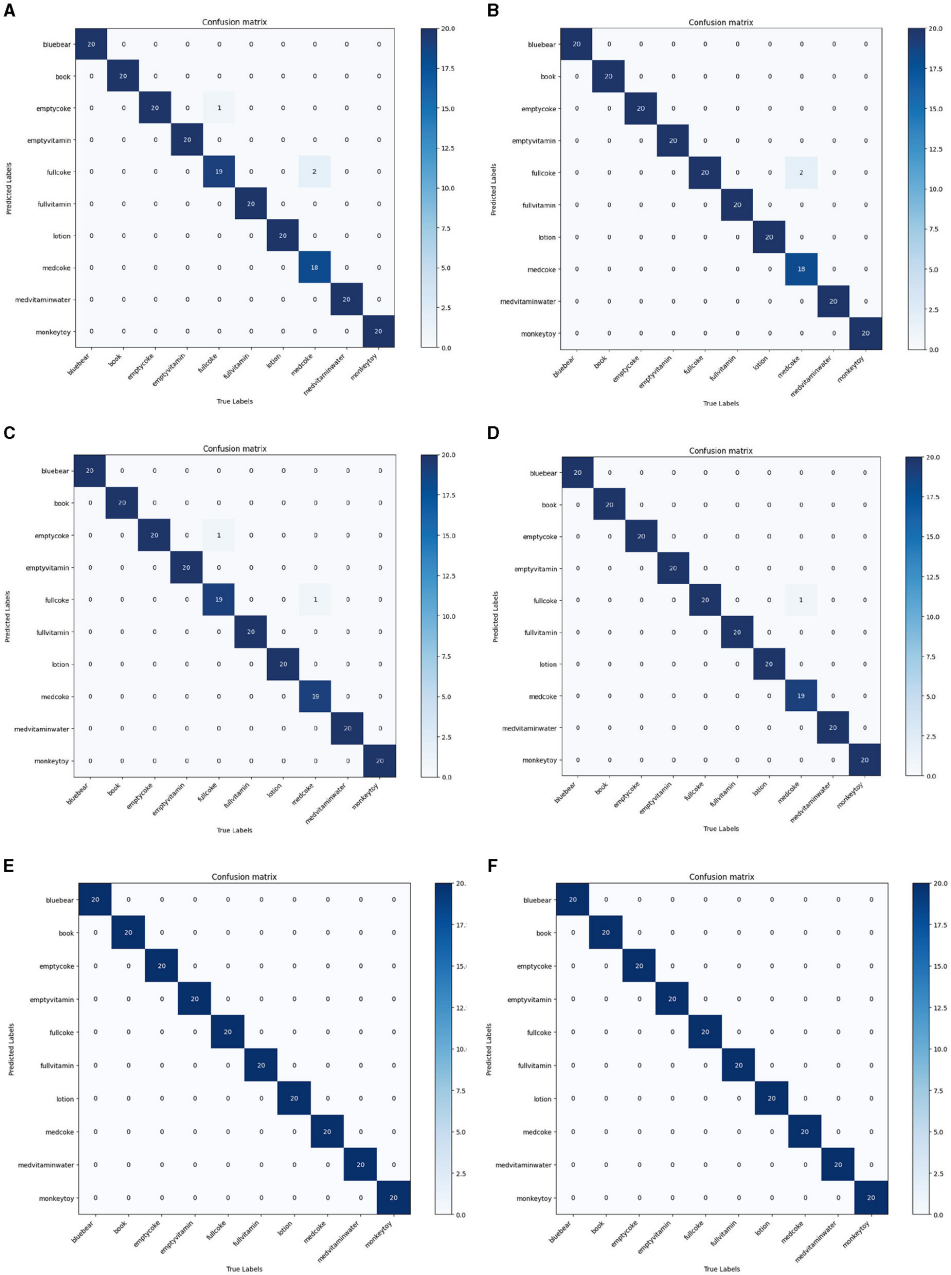
Comparisons with 5 methods in terms of confusion matrix on the iCub dataset. **(A)** DS (Soh and Demiris, 2014), **(B)** GS (Soh and Demiris, 2014), **(C)** STORK-GP (Soh et al., 2012), **(D)** STAG (Sundaram et al., 2019), **(E)** GAS-MR3D (Qian et al., 2023c), **(F)** Ours.

As shown in Table 4, our method achieved the highest top 1 score and KC. This demonstrates that our model has the best prediction accuracy and the lowest level of confusion on the MIT-STAG dataset. A comparison of the confusion matrices in Figure 7 further supports the conclusions above. As Table 5 and Figure 8 show, both our method and Qian et al. achieved a recognition accuracy of 100%. Our method and that of Qian et al. yield the highest detection accuracy and the lowest level of confusion on the iCub dataset.

**Table 4:** Comparisons with 5 methods in terms of the top 1 score (%) and KC (%) on the MIT-STAG dataset.

**Method**	**Top 1 score**	**KC**
STAG (Sundaram et al., 2019)	72.4	71.4
Smart-hand (Wang et al., 2021)	72.0	71.0
ResNet10-v1 (Zhang et al., 2021)	80.1	79.3
Tactile-ViewGCN (Sharma et al., 2022)	81.8	81.1
GAS-MR3D (Qian et al., 2023c)	88.8	88.5
Ours	91.1	90.7

**Table 5:** Comparisons with 5 methods in terms of the top 1 score (%) and KC (%) on the iCub dataset.

**Method**	**Top 1 score**	**KC**
DS (Soh and Demiris, 2014)	98.5	98.4
GS (Soh and Demiris, 2014)	98.9	98.8
STORK-GP (Soh et al., 2012)	99.3	99.2
STAG (Sundaram et al., 2019)	99.5	99.4
GAS-MR3D (Qian et al., 2023c)	100	1
Ours	100	1

In summary, our method is superior to 5 advanced methods.

## 5 Conclusion

A novel LGR-18 network is proposed to address the problem that the current TOR models cannot simultaneously extract local and global features very well. The LGR-18 network consists primarily of multiple traditional 1D and 2D convolution kernels and LGC blocks, which is proposed in this paper. The LGC block is formed by combining LC and GC modules through residual connections. The LC module mainly utilizes a temporal shift operation and a 2D convolution to extract local spatiotemporal features. The GC module extracts global spatiotemporal features by fusing multiple 1D and 2D convolutions which can expand the receptive field in temporal and spatial dimensions. In this paper, we utilize the LGR-18 network to extract local and global spatiotemporal features while mitigating the issue of large parameter in existing 3D CNN models. Ablation studies verify the validity of the LC module, GC module, and LGC block. A comprehensive quantitative comparison between our method and 5 advanced methods on the MIT-STAG and iCub datasets reveal the excellent capability of our method.

The future work of our team includes two parts. The first part is combining our method with video based object detection method, and the another part is deploying our method on more robots.

## Data availability statement

Publicly available datasets were analyzed in this study. The datasets for this study can be found in the http://humangrap.io and https:/github.com/clear-nus/icub_grasp_dataset.

## Author contributions

XQ: Writing – original draft, Writing – review & editing. WD: Writing – original draft. WW: Writing – review & editing. YL: Writing – review & editing. LJ: Writing – review & editing.

## References

[B1] Bottcher W. Machado P. Lama N. McGinnity T. (2021). “Object recognition for robotics from tactile time series data utilising different neural network architectures,” in 2021 International Joint Conference on Neural Networks (IJCNN), 1–8. 10.1109/IJCNN52387.2021.9533388

[B2] Cao L. Sun F. Liu X. Huang W. Kotagiri R. Li H. (2018). End-to-end convnet for tactile recognition using residual orthogonal tiling and pyramid convolution ensemble. Cogn. Comput. 10, 718–736. 10.1007/s12559-018-9568-7

[B3] Carreira J. Zisserman A. (2017). “Quo vadis, action recognition? A new model and the kinetics dataset,” in Proceedings of the IEEE Conference on Computer Vision and Pattern Recognition, 6299–6308. 10.1109/CVPR.2017.502

[B4] Carvalho H. N.d Castro L. P. Rego T. G. D. Filho T. M. S. Barbosa Y.. (2022). “Evaluating data representations for object recognition during pick-and-place manipulation tasks,” in 2022 IEEE International Systems Conference (SysCon), 1–6. 10.1109/SysCon53536.2022.9773911

[B5] Chung J. Lim H. Lim M. Cha Y. (2020). Object classification based on piezoelectric actuator-sensor pair on robot hand using neural network. Smart Mater. Struct. 29:105020. 10.1088/1361-665X/aba540

[B6] Gandarias J. M. Gómez-de Gabriel J. M. García-Cerezo A. (2017). “Human and object recognition with a high-resolution tactile sensor,” in 2017 IEEE Sensors (IEEE), 1–3. 10.1109/ICSENS.2017.8234203

[B7] Gao M. Zhang B. Wang L. (2021). “A dynamic priority packet scheduling scheme for post-disaster uav-assisted mobile ad hoc network,” in 2021 IEEE Wireless Communications and Networking Conference (WCNC) (IEEE), 1–6. 10.1109/WCNC49053.2021.9417537

[B8] Hara K. Kataoka H. Satoh Y. (2018). “Can spatiotemporal 3D cnns retrace the history of 2D cnns and imagenet?” in Proceedings of the IEEE Conference on Computer Vision and Pattern Recognition, 6546–6555. 10.1109/CVPR.2018.00685

[B9] Huo Y. Qian X. Li C. Wang W. (2023). Multiple instance complementary detection and difficulty evaluation for weakly supervised object detection in remote sensing images. IEEE Geosci. Rem. Sens. Lett. 20, 1–5. 10.1109/LGRS.2023.3283403

[B10] Ibrahim A. Ali H. H. Hassan M. H. Valle M. (2022). “Convolutional neural networks based tactile object recognition for tactile sensing system,” in Applications in Electronics Pervading Industry, Environment and Society: APPLEPIES 2021 (Springer), 280–285. 10.1007/978-3-030-95498-7_39

[B11] Kirby E. Zenha R. Jamone L. (2022). Comparing single touch to dynamic exploratory procedures for robotic tactile object recognition. IEEE Robot. Autom. Lett. 7, 4252–4258. 10.1109/LRA.2022.3151261

[B12] Li L. Yao X. Wang X. Hong D. Cheng G. Han J. (2023). Robust few-shot aerial image object detection via unbiased proposals filtration. IEEE Trans. Geosci. Rem. Sens. 61, 1–11. 10.1109/TGRS.2023.3300071

[B13] Liu H. Qin J. Sun F. Guo D. (2016). Extreme kernel sparse learning for tactile object recognition. IEEE Trans. Cybern. 47, 4509–4520. 10.1109/TCYB.2016.2614809 27775546

[B14] Liu Y. Bao R. Tao J. Li J. Dong M. Pan C. (2020). Recent progress in tactile sensors and their applications in intelligent systems. Sci. Bull. 65, 70–88. 10.1016/j.scib.2019.10.021 36659072

[B15] Lu X. Sun D. Yin H. Xu H. Yan Y. Wu C.. (2023). 3-D tactile-based object recognition for robot hands using force-sensitive and bend sensor arrays. IEEE Trans. Cogn. Dev. Syst. 15, 1645–1655. 10.1109/TCDS.2022.3215021

[B16] Lv Z. Huang H. Sun W. Lei T. Benediktsson J. A. Li J. (2023a). Novel enhanced unet for change detection using multimodal remote sensing image. IEEE Geosci. Rem. Sens. Lett. 20, 1–5. 10.1109/LGRS.2023.3325439

[B17] Lv Z. Liu J. Sun W. Lei T. Benediktsson J. A. Jia X. (2023b). Hierarchical attention feature fusion-based network for land cover change detection with homogeneous and heterogeneous remote sensing images. IEEE Trans. Geosci. Rem. Sens. 61, 1–15. 10.1109/TGRS.2023.3334521

[B18] Lv Z. Zhang M. Sun W. Benediktsson J. A. Lei T. Falco N. (2023c). Spatial-contextual information utilization framework for land cover change detection with hyperspectral remote sensed images. IEEE Trans. Geosci. Rem. Sens. 61, 1–11. 10.1109/TGRS.2023.3336791

[B19] Philippe F. Schacher L. Adolphe D. C. Dacremont C. (2004). Tactile feeling: sensory analysis applied to textile goods. Textile Res. J. 74, 1066–1072. 10.1177/004051750407401207

[B20] Qian X. Huo Y. Cheng G. Gao C. Yao X. Wang W. (2023a). Mining high-quality pseudoinstance soft labels for weakly supervised object detection in remote sensing images. IEEE Trans. Geosci. Rem. Sens. 61, 1–15. 10.1109/TGRS.2023.3266838

[B21] Qian X. Li C. Wang W. Yao X. Cheng G. (2023b). Semantic segmentation guided pseudo label mining and instance re-detection for weakly supervised object detection in remote sensing images. Int. J. Appl. Earth Observ. Geoinf. 119:103301. 10.1016/j.jag.2023.103301

[B22] Qian X. Lin S. Cheng G. Yao X. Ren H. Wang W. (2020). Object detection in remote sensing images based on improved bounding box regression and multi-level features fusion. Rem. Sens. 12:143. 10.3390/rs12010143

[B23] Qian X. Meng J. Wang W. Jiang L. (2023c). Gradient adaptive sampling and multiple temporal scale 3d cnns for tactile object recognition. Front. Neurorob. 17:1159168. 10.3389/fnbot.2023.1159168 37180284 PMC10169613

[B24] Qian X. Wang C. Li C. Li Z. Zeng L. Wang W.. (2023d). Multiscale image splitting based feature enhancement and instance difficulty aware training for weakly supervised object detection in remote sensing images. IEEE J. Select. Topics Appl. Earth Observ. Rem. Sens. 16, 7497–7506. 10.1109/JSTARS.2023.3304411

[B25] Qian X. Wu B. Cheng G. Yao X. Wang W. Han J. (2023e). Building a bridge of bounding box regression between oriented and horizontal object detection in remote sensing images. IEEE Trans. Geosci. Rem. Sens. 61, 1–9. 10.1109/TGRS.2023.3256373

[B26] Qian X. Zeng Y. Wang W. Zhang Q. (2023f). Co-saliency detection guided by group weakly supervised learning. IEEE Trans. Multim. 25, 1810–1818. 10.1109/TMM.2022.3167805

[B27] Sharma M. (2022). Tactile-viewgcn: Learning shape descriptor from tactile data using graph convolutional network. arXiv preprint arXiv:2203.06183.

[B28] Soh H. Demiris Y. (2014). Incrementally learning objects by touch: online discriminative and generative models for tactile-based recognition. IEEE Trans. Hapt. 7, 512–525. 10.1109/TOH.2014.2326159 25532151

[B29] Soh H. Su Y. Demiris Y. (2012). Online spatio-temporal gaussian process experts with application to tactile classification,” in *2012 IEEE/RSJ International Conference on Intelligent Robots and Systems* (IEEE), 4489–4496. 10.1109/IROS.2012.6385992

[B30] Soomro K. Zamir A. Shah M. (2012). Ucf101: a dataset of 101 human actions classes from videos in the wild. arXiv, abs/1212.0402.

[B31] Sundaram S. Kellnhofer P. Li Y. Zhu J.-Y. Torralba A. Matusik W. (2019). Learning the signatures of the human grasp using a scalable tactile glove. Nature 569, 698–702. 10.1038/s41586-019-1234-z 31142856

[B32] Wang X. Geiger F. Niculescu V. Magno M. Benini L. (2021). “Smarthand: towards embedded smart hands for prosthetic and robotic applications,” in 2021 IEEE Sensors Applications Symposium (SAS) (IEEE), 1–6. 10.1109/SAS51076.2021.9530050

[B33] Wu Y. Liu Y. Zhou Y. Man Q. Hu C. Asghar W.. (2018). A skin-inspired tactile sensor for smart prosthetics. Sci. Robot. 3:eaat0429. 10.1126/scirobotics.aat0429 33141753

[B34] Xie X. Cheng G. Feng X. Yao X. Qian X. Han J. (2024). Attention erasing and instance sampling for weakly supervised object detection. IEEE Trans. Geosci. Rem. Sens. 62, 1–10. 10.1109/TGRS.2023.3339956

[B35] Yi Z. Xu T. Shang W. Li W. Wu X. (2022). Genetic algorithm-based ensemble hybrid sparse elm for grasp stability recognition with multimodal tactile signals. IEEE Trans. Industr. Electr. 70, 2790–2799. 10.1109/TIE.2022.3170631

[B36] Zhang X. Li S. Yang J. Bai Q. Wang Y. Shen M.. (2021). Target classification method of tactile perception data with deep learning. Entropy 23:1537. 10.3390/e23111537 34828235 PMC8619335

